# Follow up of patients who start treatment with antidepressants: treatment satisfaction, treatment compliance, efficacy and safety

**DOI:** 10.1186/1471-244X-13-65

**Published:** 2013-02-20

**Authors:** Jesús López-Torres, Ignacio Párraga, José M Del Campo, Alejandro Villena

**Affiliations:** 1 Research Unit, Primary Care Head Office of Albacete, Health Care Service of Castilla-La Mancha, Marqués de Villores 6-8, Albacete, 02001, Spain; 2 Almansa Health Centre, Health Care Service of Castilla-La Mancha, C/San Juan s/n, 02640 Almansa, Albacete, Spain; 3 Albacete Area VB Health Centre, Health Care Service of Castilla-La Mancha, C/Macedonio Jiménez, s/n, Albacete, 02006, Spain

## Abstract

**Background:**

Measuring satisfaction with treatment has proved useful to ascertain the treatment features that are most important to the patients, and to explain increased treatment compliance. However, there are few studies that relate satisfaction to other clinical or self-perceived health status indicators. Recent studies have shown the close relationship between satisfaction with treatment, treatment compliance, and effectiveness. This study attempts to design and validate a scale to evaluate satisfaction with antidepressant drug therapy, assess treatment compliance (self-reported, validated questionnaire, drug accountability and electronic monitorization system), assess efficacy in reducing depressive symptoms and safety in patients who initiate antidepressant drug therapy, as well as to establish predictors of satisfaction, compliance and effectiveness with these drugs.

**Methods/design:**

This is an observational longitudinal study with a cohort of adults initiating treatment with antidepressant drugs. A multi-centre study will be performed in which 20 Primary Care practices from Castilla-La Mancha are expected to participate. An initial interview and follow-up visits at 15 days, 1, 3, 6, 9 and 12 months will be conducted with all study participants. 706 subjects will be studied (95% confidence interval, precision ± 3%, expected rate of non-compliance 50%, expected non-responders and lost to follow up rate 15%). The following measurements will be performed: development and validation of a scale of satisfaction with antidepressant therapy, participant and antidepressant characteristics, treatment compliance evaluation (Haynes-Sackett Test, Morisky-Green Test, drug accountability and Medication Event Monitoring System), depression symptom reduction (Hamilton Depression Rating Scale and Montgomery-Asberg Depression Rating Scale), observation of adverse effects, and beliefs about treatment (The Beliefs about Medicines Questionnaire).

**Discussion:**

Antidepressant drugs are an extraordinarily important therapeutic group in the pharmacy composition; economic repercussions and social impact associated to their use is clear. Despite their well-established efficacy in clinical trials, treatment non-compliance is a major obstacle to their effectiveness in clinical practice. The proposed study brings about useful conclusions to improve the results of these drugs. Additionally, devising a scale specifically designed to evaluate satisfaction with antidepressant treatment could be of interest in healthcare outcomes research.

## Background

### Use of antidepressant drugs

Since their introduction, the prescription of novel antidepressant drugs (ADs) has increased dramatically [1]. The change in the psychiatric care model, now incorporated into the rest of specialties, within primary care (PC) and closer to the patients, has been associated with an increase in the diagnoses of depression [2] as well as the indication for antidepressants –which are used to treat depression and other disorders in 36% and 64% of cases, respectively [3].

Treatment with ADs is initiated in the PC setting in 76.36% of cases. A study performed to ascertain the situation of depression-related healthcare in Spain [4] showed that most PC physicians (95%) and psychiatrists (99%) prescribe drug treatments to all their depressed patients. Both, PC physicians and psychiatrists, mostly prescribe selective serotonin reuptake inhibitors (SSRIs) as first line drugs (93% of both samples). While SSRIs are considered to be the most effective antidepressant group by PC physicians, psychiatrists rather consider tricyclic antidepressants to be the most effective.

As a consequence of the dramatic increase in their prescription, antidepressants have become the third largest pharmaceutical class sold in the year 2009 –only surpassed by anti-ulcer drugs and hypolipemiants– resulting in notably increased healthcare expenditures [5]. The actual clinical impact of this increase is presently under discussion, as is the possible abuse of these drugs in doubtful indications or for indefinite periods of time, again with respect to the impact on pharmaceutical expenditures. On the other hand, a study where drug consumption and treatment duration were analyzed [6] showed that a high percentage of individuals undergo less than 4 months of treatment, which contradicts the current recommendations regarding antidepressant drugs.

As to the use of ADs, a consumption prevalence rate of 7.4% was reported in a general population study performed in Germany [7]. Also, in another study conducted in the US [8], 21.5% of the elderly population showed significant depressive symptoms, and ADs were used in 7.5% of cases.

### Effectiveness and safety of antidepressant drugs

While ADs can be effective if well prescribed, almost 50% of prescriptions issued by primary care centres are aimed to treat subjects without a clear psychiatric diagnosis [9]. In addition, the ability to tolerate frustration seems to be progressively declining in the most developed societies, and drugs are frequently sought as a solution to personal and social everyday problems [10]. Other than an appropriate prescription, other aspects such as doses used, early medication discontinuation, and lack of follow-up by the family doctor must be considered.

Nowadays, the efficacy of ADs is very well established. However, even the most optimistic clinical trials yield efficacy rates of 70% and 40% for antidepressant and placebo, respectively [11]. While ADs have shown to be more effective than placebo in the treatment of major depression, their efficacy in the minor forms of the disorder have not been shown [12]. The systematic review performed by Kirsch [13] claims that only in case of severe disorders is there a difference between antidepressants and placebo. Another recent review only found small differences between the effects of antidepressants and placebo [14]. It has been argued that mild depression – which in the past frequently went undetected by the primary care physician– show a similar course regardless of whether they are or are not identified. In this sense, part of the extraordinary increase in AD prescription can be attributed to this group of patients, who previously failed to receive treatment for an indication without sufficient evidence of efficacy data available [15]. In fact, in PC up to one fourth of patients with a prescription for ADs collect their prescriptions only once and do not continue treatment.

In general, the efficacy of all first-line ADs is similar, and therefore the antidepressant selection process is determined by the incidence and severity of adverse effects, interactions, dosing convenience, patient characteristics (age and concomitant diseases) or their beliefs about treatment [16] and cost. The differences between the different ADs mainly depend on their pharmacokinetic characteristics and side effects. A meta-analysis [17] of the efficacy and tolerability of SSRIs vs tricyclic antidepressants in the treatment of depression in the primary care setting showed evidence of similar short-term efficacy, although tolerability for SSRIs was better.

### Treatment compliance

In view of the safety of current antidepressants, clinical guidelines have become more and more conservative in determining the maintenance period of ADs [18]. Thus, it is recommended that treatment be maintained for at least 1 year before starting gradual withdrawal, or to continue for at least 6 months after obtaining a response in depressive disorders [19]. Treatment compliance, however, is low. Non-compliance is associated to an important risk of recurrence of depressive disorders and evolution to chronicity [20,21]. In addition, therapeutic non-compliance has also been associated with increased morbimortality as well as mid- and long-term costs [22]. Several studies have shown high rates of treatment dropout, ranging from 30-40% during the first months [23,24] up to 50-60% during the maintenance period [21].

A study performed to assess adherence to antidepressant treatment reported correct intakes of prescribed doses after 1 month of therapy in 82% of cases. This percentage fell to 69% after 3 months [25]. For most ADs, overall efficacy is limited by long-term treatment compliance. However, although is really difficult to know whether a patient actually takes his/her pills as prescribed, this is a key factor to achieve and maintain improvement. This is well illustrated by the fact that depression has been included by the WHO in a monograph on “adherence” to treatment for chronic diseases such as hypertension, asthma, diabetes or epilepsy [26]. Other reasons for treatment dropout have also been established: 23% of cases due to adverse effects, 10% fear of drug dependence or inappropriate treatment, 10% for considering subjectively that treatment is ineffective, and 9% for reasons attributed to healthcare providers, who erroneously tell the patient to discontinue treatment. Several measures have been suggested to increase treatment adherence, such as using better tolerated drugs, simplest drug administration schedules, help from relatives, pill reminders, psychoeducation, etc.

Estimating treatment compliance is a particularly complex task that can involve patient self-reports, drug accountability records, computerized packaging systems, or plasma level determinations. Some of the studies have even compared the validity of the different methods of measurement. George et al. [27] compared 4 evaluation methods in depressed patients treated in primary care and showed that an electronic medication event monitoring system (MEMS) for daily pill count was the most reliable method, followed by simple patient self-reports, while plasma level determination was cumbersome and quite inconvenient. In short, no simple, convenient, fully reliable gold standard system is available [28].

Also to be acknowledged are factors related to good or bad compliance, such as dose frequency, medication type, etc. Relating to dose frequency, studies like that by Myers and Branthwaite [29] failed to point out differences between once vs three times daily dosages, but compliance was clearly better when the patient him/herself decided on his/her own dosage regimen. Also interesting is the drug type, where it has been suggested that SSRIs are far better than tricyclics [30,31], with a lower percentage of adverse effects but a higher dropout rate for lack of efficacy. Other meta-analyses discussing this subject have suggested that compliance depends on the involvement of patients and healthcare providers in follow-up tasks, rather than the drug type itself, [32]. There is, however, a limited number of studies on factors related to treatment non-compliance with these drugs.

Other variables associated with better compliance include psychiatrist follow-up or consumption of other medications (excluding psychoactive drugs) [33]. In contrast, factors that contribute to non-compliance include adverse effects on sex life, self-perception of medication as being ineffective, female sex, low educational level [34], psychiatric comorbidity [21,35] and poor social support or functional limitations in the elderly [36]. The studies on AD compliance predictors frequently focus on a very limited range of clinical and sociodemographic characteristics and so inconsistent results are sometimes obtained.

### Satisfaction with antidepressant treatment

Satisfaction with treatment is part of the satisfaction with the healthcare received [37]. It can be defined as the evaluation of the process of drug treatment intake and its outcome [38]. Another definition based on psychosocial theories emphasizes the importance of the patient’s attitude toward the different dimensions involved in his/her treatment, and compares patient expectations with actual facts [39]. Satisfaction is measured by means of standard, validated questionnaires completed by the patients who receive treatment. Satisfaction with treatment is expressed in by a variety of dimensions that depend on the type of treatment [37]. Most frequent dimensions include: symptom relief, drug tolerability profile (some treatments may be perceived by the patient as “worse than the disease”), convenience of administration, etc. Satisfaction with the impact of treatment on the dimensions involved in health-related quality of life (HRQOL), such as physical or psychological functions, is also included. Finally, general satisfaction with treatment, and well as whether the patient would recommend it to other patients with the same health problem, must be evaluated.

Given that a chronic patient is to take medication for a long period of time, measuring satisfaction with treatment in case of chronic therapies of diseases such as depression is particularly recommended. Satisfaction with treatment may be related to treatment compliance. A more satisfied patient takes his/her medication properly for the prescribed length of time and thus contributes to the desired therapeutic effect. In addition, satisfaction with treatment must relate to the patient’s preferences. Indeed, when given the opportunity to try different treatments, the patient will choose the most satisfying one.

### Rationale for the study

The clinical effectiveness of any therapy is determined by the evaluation of (usually objective) clinical parameters of the studied health condition. Hence, treatment success is mainly based on the improvement or disappearance of symptoms as well as on an objective assessment of the physical or mental status by different measuring methods. Health Outcomes Research complements clinical indicators with other measurements that are more relevant to the patient, such as health status and health-related quality of life (HRQoL).

Studies relating satisfaction to other clinical or self-perceived health indicators are still scarce. The frequently close relationship between satisfaction with treatment, treatment compliance and effectiveness has already been shown. The strength of the relationship between satisfaction, preferences and clinical or self-perceived measures may depend on the characteristics of both the disease and its treatment. Aspects that are most relevant to an asymptomatic patient with a chronic disease, such as hypertension, will differ from those that are most relevant to patient with a chronic symptomatic disease such as depression. Convenient drug regimens may be preferred in the former, while other aspects such as achieving symptom control as soon as possible may be more important in the latter.

Few questionnaires on satisfaction with treatment have been developed so far. Over recent years, some have been developed to address health conditions such as diabetes, acne, erectile dysfunction, rheumatoid arthritis, arthrosis, pain or chronic diseases in general [40-44]. However, no questionnaires on patient satisfaction and preferences relating to depression treatment are currently available, and this aspect does bear a considerable impact on the patient’s daily activities and HRQoL.

Research on satisfaction with treatment is still in its early days, and many issues remain to be known and analyzed in this field. Chronic processes in which therapeutic changes are frequent, increased numbers of drug products marketed for the same disorder, and broader access to information by the patient, all make him/her wish to participate more in the decisions about his/her treatment and the healthcare provider give more consideration to the patient’s opinion [37].

It is reasonable to assume that the prescription and use of the most successful drug products according to patient satisfaction and preferences should eventually lead to better treatment compliance and hence better effectiveness and more benefit.

Long-term follow-up studies are required to help physicians opt for the more effective and better tolerated treatment options. This information should preferably be generated by the healthcare providers themselves. Publications studying the evaluation of antidepressant treatment duration in Spain are scarce, and more studies in the primary care setting are needed to clarify possible differences in response among different antidepressants with different mechanisms of action. Consequently, the conduction of studies evaluating the clinical effectiveness of different antidepressant therapies, the adherence to them and their safety under actual conditions of use, should be prioritized.

### Study objectives

1. To develop and validate a specific tool to assess satisfaction with antidepressant drug therapy and determine its convergent validity with respect to the main measurements conceptually related to the assessed construct: clinical effectiveness, treatment-related beliefs and expectations, treatment compliance, and tolerability.

2. To establish both clinical and sociodemographic predicting factors of greater satisfaction with antidepressant drug therapy.

3. To assess adherence to antidepressant drug therapy and hence treatment compliance (self-reported, using a validated questionnaire, by drug accountability and using an electronic monitoring system) in patients who initiate antidepressant drug therapy.

4. To analyze factors determining non-compliance from both a clinical (concurrent diseases, consumption of other drug products, etc.) and sociodemographic viewpoint.

5. To evaluate the effectiveness of antidepressant drugs on the remission of depressive symptoms by using previously standardized instruments to evaluate symptom severity and treatment-induced changes (Hamilton Depression Rating Scale and Montgomery-Asberg Depression Rating Scale).

6. To evaluate drug-related safety by describing adverse effects of the different antidepressant drugs.

## Methods

### Design

This is a longitudinal observational study where a cohort of adult patients initiating treatment with antidepressants will be assessed after 15 days, 1, 3, 6, 9 and 12 months.

### Study sites and subjects

This is a multi-centre study involving 20 Primary Care centres distributed among the eight healthcare areas of Castilla-La Mancha. This autonomous community has a population of 1,977,304 inhabitants, that is, 4.32% of the Spanish population [45].

Study subjects will include patients aged 18 years and older, undergoing therapy with newly prescribed antidepressants. The accessible population will include users of the Family Care Medicine surgeries of the participating Health Centres.

Inclusion criteria: patients aged 18 years and older initiating treatment with antidepressants prescribed by the family care doctor (GP), or a psychiatrist, or another specialist, who give their consent to participate in the study once properly informed about its objectives.

Exclusion criteria: subjects with poor intellectual performance that proves insufficient for them to collaborate in the study; drug dependence; severe organic, mobility-hindering diseases; and use of any antidepressant within 3 months prior to the initiation of the study.

### Sample size

In order to achieve a 95% confidence level, a precision of ± 4%, and an expected overall rate of non-compliance with antidepressant treatment of 50% (30-40% during the first months and 50–60% during maintenance treatment), a total of 600 patients initiating treatment with antidepressant drugs for the study period will need to be included. Considering a 15% rate of non-responders and losses to follow-up, a final sample size of 706 participants is obtained. These will be selected by consecutive sampling at the surgeries of the participating health centres.

### Study variables

Participant characteristics: age, sex, level of education, occupation-based social class, type of coexistence, marital status, type of funding for drug products provision (active or pensioner), existence or non-existence of temporary disability in case of active workers (throughout the observation period) and reason for attending, as well as visits to general medicine surgeries in the previous 3 months.

Antidepressant drugs: antidepressant type (NO6A Group under the Anatomical Therapeutic Chemical Classification [ATC]), dosage regimen, main reason for prescription, prescribing physician (family doctor, psychiatrist or other specialist), adverse effects, previous consumption of antidepressants, reasons stated by the patient to explain why he/she does not take regularly or abandons his/her medication.

Health status: health problems (International Classification of Primary Care - ICPC – 2 – WONCA), consumption of other drug products (ATC Classification), history of depressive disorders in the patient’s medical records and possible concomitant psychotherapeutic treatments.

Adherence to medication: self-reported compliance (Haynes-Sackett Test), validated questionnaire on extent of compliance based on clinical interview (Morisky-Green Test) [46], drug accountability (percentage of treatment compliance: number of presumably consumed pills/total number of pills that should have been consumed x 100). In a sub sample of 50 randomly selected patients, compliance will be evaluated by means of the Medication Event Monitoring System (MEMS) (AARDEX, Switzerland).

Evaluation of depressive symptoms: depressive symptom severity by means of the Hamilton Depression Rating Scale [47] (17-item version), adapted to Spanish and validated by Ramos-Brieva and Cordero in 1986 [48]; change in the intensity of depressive symptoms as a result of pharmacological intervention by means of the Montgomery-Asberg Depression Rating Scale (MADRS) [49], adapted to Spanish by Conde and Franch in 1984 and validated by Martínez R et al. in 1991 [50].

Beliefs about treatment, evaluated by means of The Beliefs about Medicines Questionnaire (BMQ), validated in Spain by Beléndez Vázquez in 2007 [51], and degree of expectations fulfillment (questions have 5 response options).

### Satisfaction scale development and validation

The scale will be developed as follows: firstly, a thorough review of the medical literature will be conducted to identify all clinical manifestations of depressive patients, all aspects related to the antidepressant treatment and the contents of other satisfaction scales developed for other conditions. As a result of this review, a first version of the satisfaction scale will be prepared. Question selection will be done according to interpretability, literacy level, item extent, validity, discriminative ability and homogeneity.

Then, a meeting will be held with a group of experts in the disease (psychiatrists and family doctors) to evaluate whether clinical manifestations as well as aspects that are key to the patient’s satisfaction with depression treatment are properly reflected in the questions. Question content will be analyzed in terms of understanding, format and applicability. Subsequent to this review, modifications on the initial scale may be introduced and questions may be added, this leading to a second version of the scale.

The second version of the scale will be administered to 15 patients undergoing antidepressant therapy. These interviews will be aimed at validating the scale content from the patient’s viewpoint. After scale administration, a survey will be conducted to ascertain the patient’s opinion on the level of understanding and applicability of each question. This is how the final version of the scale will be obtained. The questions included in this final version will refer to specific aspects of treatment, such as effectiveness (symptom control, absence of discomfort and convenience) and daily life aspects in which patients find particular improvement. The final scale will include Likert-type questions with 5 response options, where subjects will be able to express to what extent they agree with the different assertions stated in the scale (from “fully agree” to “fully disagree”).

Then, the measurement properties of the satisfaction scale will be evaluated by having all selected patients answer the questions included in the scale. Validity will be completed by further collection of beliefs on antidepressant treatments (The Beliefs about Medicines Questionnaire) as well as of patient expectations at the beginning and at each follow-up visit. Clinical effectiveness will be assessed by the scores obtained in the Hamilton Depression Rating Scale and the Montgomery-Asberg Depression Rating Scale.

The satisfaction scale will be re-administered to a reduced group of patients, one week after the 1st- and 3rd-month visits. This measurement will be later used as a re-test to study instrument stability.

### Data collection and information management

Patients will be selected at primary care centres by the family care doctors who participate in the study. Once briefed on the purpose of the study and the reasons for their inclusion, patients will be addressed to the nursing station, where they will undergo a structured interview. They will be appointed for visits at 15 days, 1, 3, 6, 9 and 12 months for re-evaluation.

Data will be collected on a pre-coded questionnaire specifically designed for this study. The questionnaire will be prepared according to the following steps: selection of required information and appropriate scales (including the previously developed scale of satisfaction with antidepressant treatment), selection of question type for each variable, code and score definition, question ordering, questionnaire format design and instruction manual preparation.

A pilot test will be performed in a few individuals to establish the clarity of all questions and instructions included in the questionnaire, and the time needed to complete the interview. A standardized interview process will be used by all nursing stations for more reliability. To this end, previous training sessions will be scheduled to ensure the uniformity of data collected by the nursing staff.

In order to minimize missing data and errors, the answers will be checked by the interviewer in the presence of the interviewee, and incomplete or ambiguous questions will be corrected. Responses will then be entered in a database and reviewed periodically for incomplete or erroneous data.

The initial visit will be performed by nursing staff following prescription by the physician, and the following variables will be collected in the data collection questionnaire: social-demographic data, previous (if applicable) and present antidepressant treatment, dosage regimen, diseases and other concomitant treatments, evaluation of depressive symptoms and initial beliefs and expectations about treatment.

At the first visit, the 50 patients assigned to the MEMS system for compliance evaluation will be given the device, and its operation will be explained to them. Antidepressant prescriptions will be issued to the patients as applicable. Patients will purchase their medication at the pharmacy and will bring it back to the nurse. At the time the patient produces the medication, blisters will be removed from the box and each tablet, packed in its original packaging, will be cut out and introduced in the MEMS system.

On successive visits (at 15 days and 1, 3, 6, 9 and 12 months), also performed by the nursing staff, information will be obtained on: treatment compliance, patient-reported adverse effects, reasons stated by the patient explaining why he/she does not take regularly or abandons his/her medication, modifications in antidepressant treatment or concomitant medication, onset of new health problems, depressive symptom re-evaluation, treatment satisfaction, beliefs about treatment, and fulfilment of expectations raised by antidepressant treatment.

### Statistical analysis

Patient socio-demographic and clinical characteristics will be described by means of proportions, measures of central tendency and dispersion measures, according to the nature of the variables. Data will be summarized by means of tables and graphs, as appropriate. The corresponding 95% confidence intervals will be constructed and the response rate will be described, listing the available features of individuals who decide not to participate in the study.

Prior to evaluating the validity of the satisfaction questionnaire, feasibility will be evaluated in terms of percentage of unanswered questions. The first aspect of validity analysed will be the ceiling and floor effects of each item, defined as the percentage of patients with maximum and minimum responses, respectively. The item-total correlation (homogeneity index) will be established by measuring the correlation between the scores of each item and global scores. The Cronbach’s alpha statistic will be used to check internal consistency.

For proper construct validity, scale content will be analyzed qualitatively and checked for consistency with the theoretical concept of satisfaction with treatment. Underlying and fundamental dimensions will be explored by factor analysis with factor extraction, using principal component analysis, Varimax orthogonal rotation and Promax oblique rotation, in order to establish the different nuances raised in the questionnaire.

For content validity evaluation, the questions contained in the questionnaire will be checked for inclusion of information on all the dimensions involved in satisfaction with antidepressant treatment. To this end, questions will contemplate aspects referring to all clinical manifestations reported by depressive patients and to all aspects related with the antidepressant drugs. All questions will be supervised by clinically experienced psychiatrists and family doctors.

Because criterion validity cannot be evaluated due to lack of a gold standard of satisfaction with antidepressant treatment, only its relationship with other variables likely to be related can be evaluated, namely, fulfilment of expectations created before starting treatment, clinical effectiveness (reduction of depressive symptoms), absence of adverse effects and good therapeutic compliance. Patients with better response in terms of clinical effectiveness, better tolerability and better fulfilment of expectations raised by treatment are expected to be more satisfied with the treatment. Therefore, convergent validity will be assessed according to measurements conceptually related to the evaluated construct (Morisky-Green questionnaire, BMQ questionnaire, and Hamilton and Montgomery-Asberg Rating Scales, etc.).

Sensitivity to change will be evaluated by quantifying global score differences between visits. Change will be assessed by repeated measures ANOVA. Stability, or test-retest reliability, will be assessed in a sub sample of patients by means of the intra-class correlation coefficient (ICC) between baseline measurement and the measurement performed one week after.

The relationship between continuous variables will be analyzed with the Pearson correlation coefficient, and the relationship between two categorical variables will be analyzed with the chi-square test (after verification of the test conditions; if conditions are not met, Fisher’s exact test will be used). A *t*-test comparing the means in independent groups will be used to study the relationship between binary and quantitative variables. When assumption of normality and equality of variance are not met in the distribution of any variable, a non-parametric test will be used (Mann–Whitney *U* test for independent groups). ANOVA and Kruskal-Wallis H test will be used to compare means in more than two independent groups; the H test will be used in case of unmet conditions for ANOVA application, and in case of ordered categorical variables.

Multivariate analysis (logistic regression models) will be used to check the association of dependent variables (treatment compliance, depressive symptom reduction, onset of adverse effects and satisfaction with treatment) with their conditioning factors. Statistical adjustment will be performed and the existence of confounding factors and interaction variables will be checked. The purpose of these analyses is to estimate partial regression coefficients expressing the relevance of the different independent variables in explaining dependent variables variability. These analyses will be conducted by means of the Logistic Regression procedure of the program IBM SPSS Statistics 19.0.

### Ethics approval

This project was approved by the Research Ethics Committee of the University Hospital of Albacete on 24 July 2008.

## Discussion

The aim of this study is to address antidepressant drug prescription in the Primary Care setting by evaluating treatment compliance, clinical effectiveness to reduce depressive symptoms, safety, and treatment satisfaction. This will be done by identifying the determinants and predictors of a better result in any of these aspects by means of a longitudinal study which involves one-year follow-up of each participant.

Antidepressant drugs are an extraordinarily important therapeutic group in the pharmacy composition; economic repercussions and social impact associated to their use is clear. Despite their well-established efficacy in clinical trials, treatment non-compliance is a major obstacle to their effectiveness in clinical practice. Irregular drug intake and antidepressant treatment discontinuation have been associated with a greater risk of therapeutic failure and recurrent depressive episodes. The proposed study brings about useful conclusions to improve the results of these drugs. Indeed, the empirical identification of predictors of poorer adherence to medication would allow these predictors to be considered together with the patient during treatment, hence facilitating good compliance.

Additionally, devising a scale specifically designed to evaluate satisfaction with antidepressant treatment could be of interest in healthcare outcomes research. Thus, designing of a reliable and clinically useful instrument to evaluate treatment satisfaction will be attempted. This instrument will be subsequently validated in the target population where it is to be administered.

When preparing a scale of antidepressant treatment satisfaction, the use of closed questions may prove quite useless to obtain complex information; also, it may lead to response induction and may fail to collect important data. This modality, however, will allow response uniformity leading to easer coding. Should study losses be substantial, failing to be randomly distributed –with subjects studied not being representative of all patients treated with antidepressant therapy– a measurement bias could be present and the validity of the study would be compromised. On the other hand, the characteristics of the setting where the study will be conducted (socioeconomic status, lifestyle, etc.) may prevent the results from being directly applicable to other settings.

This being a study to be conducted simultaneously in several healthcare centres, protocol deviations or misinterpretations could lead to error. This will be prevented by study staff training and certification.

The study will adhere at all times to the following ethical principles: consent and voluntary participation, guarantee of complete privacy of information provided by the patient and restriction of all data provided by the interviewed patient to the scope of the proposed research only. Investigators will ensure that the study is conducted in full compliance with the declaration of Helsinki and according to current legislation (Royal Decree 223/2004) and to the New Code of Medical Ethics and Deontology approved by the Spanish Royal College of Physicians. The study will fully adhere to Good Clinical Practice guidelines.

## Competing interests

The authors declare that they have no competing interests.

## Authors’ contributions

JL-T, IP, JMC and AV are the investigators responsible for project design and protocol writing. JL-T and IP have participated in sample size calculation and statistical analysis planning. JL-T, IP, EG, JMC, AV, SM have contributed to study background, general design, study variable definition and adaptation to the computerized clinical record system. All authors have contributed to the preparation of the project and have read and approved the final manuscript.

## Authors’ information

Collaborating researching ADSCAMFYC Group: Fernando Andrés Pretel, Juan Miguel Armero Simarro, Rosa Armero Martínez, Francisco Javier Arribas Aguirregaviria, Candelaria Ayuso Raya, Clotilde Boix Grass, Jesús Buendía Bermejo, Silvia Darias Valenciano, Rocío Pilar Elicegui Molina, Mariano Esbri Victor, Francisco Escobar Rabadán, Ana Rus Galindo Carreño, Eva Facundo Becerra, Damarís Gómez Pimpollo, Mª Dolores González Céspedes, Enrique González Hidalgo, Carlota Ibañez Guardiola, Ángeles Lloret Callejo, Yolanda López Gallardo, Marta Lucas Pérez-Romero, Javier Lucas Pérez-Romero, Alejandro Manjavacas Garrido, Inmaculada Marín Jara, Belén Martín Águeda, Carmen Martínez Gallardo, Miriam Martínez Ramírez, Josefina Monedero La Orden, Julio Montoya Fernández, Susana Morena Rayo, Beatriz Navarro Bravo, Joseba Rabanales Sotos, Mª Dolores Retuerta García, Estrella Rivas Nieto, Adoración Romero Sáez, Julián Pedro Ruano Venceslá, Ignacio Sánchez Barranco, Mª José Simarro Herráez, Juan Manuel Téllez Lapeira, Mª Isabel Tofiño González.

## Pre-publication history

The pre-publication history for this paper can be accessed here:

http://www.biomedcentral.com/1471-244X/13/65/prepub
